# A Thirty-Year Forensic Case Report of Chronic Psychosis: Systemic Implications for Italian Forensic Psychiatry

**DOI:** 10.3390/healthcare13182302

**Published:** 2025-09-15

**Authors:** Luigi Buongiorno, Anna Margari, Gabriele Mandarelli, Roberto Catanesi, Anna Cassano, Francesco Carrieri, Lia Parente, Felice Carabellese

**Affiliations:** 1 Interdisciplinary Department of Medicine, Section of Criminology and Forensic Psychiatry, University of Bari “Aldo Moro”, 70124 Bari, Italy; 2 Department of Human Neurosciences, University of Rome “Sapienza”, Piazzale Aldo Moro 5, 00185 Rome, Italy

**Keywords:** REMS, forensic psychiatry, treatment-resistant psychosis

## Abstract

**Background:** Italy’s forensic psychiatric system has undergone major reforms over recent decades, shifting from custodial institutions to a right-based, community-oriented model. While internationally praised for its commitment to dignity, recovery, and social reintegration, this framework presupposes a degree of clinical recoverability that is not always achievable. Individuals with chronic, treatment-resistant disorders—particularly those who remain socially dangerous—pose complex challenges that strain the system. **Methods:** We report a thirty-year longitudinal case of G.D.M.: a man initially diagnosed with schizophrenia and later with chronic delusional disorder. Clinical data, forensic assessments, institutional records, and community-based rehabilitation reports were reviewed to reconstruct his clinical, criminological, and forensic trajectory. **Results:** The patient committed a femicide in early adulthood and, decades later, engaged in stalking behavior toward the victim’s family. Despite prolonged institutionalization, pharmacological treatment, multiple forensic evaluations, and rehabilitation programs, his psychopathology remained substantially unchanged. He persisted with fixed delusional ideation, exhibited minimal insight, and maintained a high level of social dangerousness. **Conclusions:** This case exemplifies structural gaps in Italy’s forensic psychiatric system, particularly the absence of stratified security levels, long-stay therapeutic facilities, and post-custodial continuity of care for individuals who do not recover. It also highlights the increasing burden placed on the Dipartimenti di Salute Mentale (DSM, Italian Mental Health Departments), which must manage both general psychiatric and high-complexity forensic cases, often without adequate resources or training. Targeted reforms—including secure long-term care pathways, systematic outcome monitoring, and clearer forensic mandates—are needed to ensure proportionate, ethical, and effective management of such cases.

## 1. Introduction

Over the past half-century, Italy has enacted one of the most radical reforms in forensic psychiatry among Western democracies. The Basaglia Law (Law 180/1978) initiated the closure of psychiatric hospitals and shifted general mental healthcare to community-based services, while forensic psychiatry remained under the jurisdiction of the Ministry of Justice, with mentally ill offenders confined in *Ospedali Psichiatrici Giudiziari* (OPGs, Forensic Psychiatric Hospitals) [1,2].

A decisive turning point occurred with the Prime Minister’s Decree of 1 April 2008, which initiated the gradual transfer of responsibility for the psychiatric care of offenders to the *Servizio Sanitario Nazionale* (SSN, Italian National Health Service), embedding the principles of dignity, recovery, and social reintegration within forensic psychiatry [1]. The full dismantling of the OPG system was mandated by Decree-Law No. 211/2011 and completed by 2015, with the creation of a decentralized, *Residenze per l’Esecuzione delle Misure di Sicurezza* (REMS, Residences for the Execution of Security Measures) [3].

REMS are healthcare facilities with a generally low security profile, though regional autonomy has led to heterogeneous standards: some units can be classified as medium security, while others provide lower levels. They operate with closed doors but without prison-like walls, armed guards, or electronic surveillance. Security is ensured mainly by architectural features and the continuous presence of health professionals (psychiatrists, nurses, educators, social workers), rather than custodial staff [3,4,5,6].

Currently, based on judicial assessments of psychiatric social dangerousness under Article 203 of the Italian Penal Code (PC), individuals deemed not criminally responsible (Art. 88 PC) or partially criminally responsible (Art. 89 CP) due to mental illness are assigned to an individualized placement pathway, which may include REMS, psychiatric rehabilitation communities, or outpatient community mental health services [1]. This stratified system, often described as a prison–REMS–community Mental Health Departments circuitry, represents the culmination of Italy’s long-standing process of deinstitutionalization in forensic psychiatry [2].

In this context, responsibility for offender care shifted from the Ministry of Justice to the National Health Service, embedding principles of dignity, recovery, and social reintegration [1,7].

This transition marked a significant cultural and institutional shift, from indefinite custodial detention to a model centered on individual rights, rehabilitation, and recovery. Italy’s forensic system has since aimed to minimize coercion, reduce institutionalization, and promote patient dignity and social reintegration [1,6,7,8,9].

While visionary, the reform presumes a level of clinical malleability that does not apply to every patient. Individuals with treatment-resistant mental disorders—particularly those whose dangerousness fluctuates below the legal threshold for custodial measures—often find no fitting therapeutic destination [9,10].

Treatment-resistant psychiatric disorders—particularly schizophrenia, bipolar disorder, personality disorders, and comorbid substance use—remain a key challenge in forensic psychiatry [11]. In fact, between 20% and 50% of individuals with severe mental illness do not respond to at least two adequate treatment trials, even when diagnostic accuracy, therapeutic optimization, and adherence are ensured [11].

These disorders—distinguishable in primary, secondary, or pseudo-resistance—undermine the core objectives of Italian forensic systems, which rely on patients’ capacity for rehabilitation and social reintegration. At the same time, treatment-resistant psychiatric disorders put into crisis the effectiveness of general psychiatry facilities, because when the latter have to deal with the long-term treatment of complex patients such as those presented in this case, they risk being completely ineffective [12].

In systems such as those of the United Kingdom or the Netherlands, graded security levels (high, medium, low) and long-stay units offer tailored pathways to accommodate long-term forensic patients [13,14].

In contrast, Italy provides a single security level with limited capacity. While the national reform process has dismantled indefinite custodial detention and promoted a shift toward recovery-oriented care, this model has also generated unintended consequences.

In the past, the absence of alternative structures contributed to the phenomenon of ‘*ergastolo bianco*’ (‘white life sentence’), whereby individuals deemed socially dangerous could be detained in forensic facilities well beyond the term of their criminal sentence [15]. Although this practice is no longer legally permitted [1], structural limitations in facility availability and care planning persist, continuing to pose significant challenges. Some patients are discharged into clinically inadequate settings, while others are retained beyond necessity due to a lack of appropriate placement options [9].

This raises concerns regarding the protection of individual rights and the risk that such structural gaps may undermine the very principles of dignity, recovery, and social reintegration on which the Italian forensic system and general psychiatry care are founded. A gap that also has repercussions for the general psychiatric care system, which reveals consequential critical issues that cannot be resolved with the procedures dictated by the current regulatory framework. In this regard, we recall that Law 81/2014 has, in fact, attributed to *Dipartimenti di Salute Mentale* (DSM, Italian Mental Health Departments) the coordination of care, assistance and rehabilitation recovery activities for both patients with mental problems who do not commit crimes and those who commit crimes, according to the same community care models [1,3].

This case report follows the three-decade forensic and clinical trajectory of a man with chronic psychosis and a history of homicide and stalking behavior. Despite multiple evaluations, institutional placements, and therapeutic attempts, his psychopathology remained substantially unchanged. His case exemplifies the critical gap in Italy’s forensic psychiatric framework: the absence of structural provisions for managing unrecoverable psychiatric conditions beyond formal dangerousness [16].

This case is presented not only for its clinical trajectory but also as an illustrative example of systemic challenges within Italian forensic psychiatry, thereby integrating individual-level observations with broader structural considerations.

## 2. Case Report

G.D.M., an Italian male born in the early 1960s, was raised in a structurally intact family as the second of four siblings. He was born after an uncomplicated delivery, breastfed, and did not report early developmental abnormalities. Nonetheless, from early childhood, he demonstrated a strong preference for solitary activities and a marked lack of interest in forming close relationships. He described himself retrospectively as *‘a child with no friends,’* and his early schooling was characterized by social isolation.

Around the age of 15–16, a marked decline in school performance was observed, accompanied by growing difficulties in social adjustment. He attributed this drop to self-perceived memory problems, which he linked to masturbation. At approximately 19–20 years of age, he obtained a high school diploma after repeating a year and with minimal grades. He subsequently attempted to enroll in a professional training course for nurses, but was not admitted, an event he perceived as a significant injustice. After this episode, he did not pursue further studies. His occupational history includes employment as a school janitor through a disability-related hiring program. However, his contract was terminated during the probationary period due to behavioral concerns. At the age of 22, inappropriate interactions with a 13-year-old girl raised serious concerns, leading to intervention by the girl’s father and a negative report from the school. Following this incident, he was transferred to another institute, where at the age of 23, he engaged in a similar episode involving a 15-year-old girl. This relationship lasted approximately two years and was associated with significant functional impairment. He also reported experiencing intense anger and violent ideation, stating: ‘*She won; she did not want a relationship with me, and she did not have one. I was very angry; I almost killed her, but decided she was not worth it.*’

From the age of 18, he reported a constant search for ‘*a woman for me, someone pretty,*’ and began attending church regularly as his main social activity. During this period, he encountered S.S., a young devout Catholic student, and became infatuated. After declaring his feelings and being rejected, he was warned by local authorities not to return to her town. Nonetheless, his obsession with S.S. persisted for nearly three years, severely impairing other areas of life functioning. At the time, anti-stalking laws in Italy were not yet in place, and despite repeated complaints, the legal system proved ineffective in addressing the issue. As a result, the victim was forced to always be accompanied in public.

During this period, at the age of 30, he was diagnosed with schizophrenia by the local mental health service. Approximately one year after the initial complaints, G.D.M. assaulted the victim physically, leading to further police involvement and the initiation of antipsychotic treatment. The treatment was resisted by both the patient and his family and was discontinued early due to reported side effects.

Two years later, G.D.M. delivered a threatening note to S.S. stating: ‘*You will be mine or no one’s, not even God’s.*’ The victim responded: ‘*Know that whatever happens to me, I have chosen God.*’ The final episode occurred shortly after: G.D.M. assaulted S.S. with a kitchen knife near her residence. The victim sustained multiple stab wounds to the neck and chest and died shortly after hospital admission. At the time of the offense, G.D.M. was 33 years old, and the victim was 21.

A first forensic psychiatric evaluation, ordered by the court in 1991 after the homicide, diagnosed G.D.M. with chronic schizophrenia, characterized by systematized delusions, disorganized associative processes, and affective blunting. At the time of the offense, he exhibited only partial awareness of the nature and wrongfulness of his actions. Psychometric testing, including the Rorschach, revealed marked negativism, impaired reality testing, and symbolic disorganization. He was deemed entirely mentally incapacitated and highly socially dangerous and was admitted to a forensic psychiatric hospital.

During institutionalization, he continued to receive antipsychotic treatment. According to the available records, the therapeutic plan initially included haloperidol decanoate (50 mg every two weeks), and from 2002 onward, olanzapine (15 mg/day). These were the only regimens systematically documented, although the use of other treatments during the long-term course cannot be ruled out. Throughout his institutionalization, the patient maintained antipsychotic therapy. The available records show that the treatment started with haloperidol decanoate (50 mg biweekly), and from 2002 onward, olanzapine (15 mg/day) was administered. Considering the documented clinical stability over time and the mild fluctuations in symptom severity and frequency, the treating physicians made only minimal adjustments to the pharmacological regimen.

No significant improvements were observed over the years. Notably, after the cloning of Dolly the sheep in 1996, G.D.M. developed a delusional plan to ‘resurrect’ S.S. by cloning her from maternal cells. He sent letters to her family outlining this plan and offering himself as ‘guardian’ of the new being (see Supplementary Figure S1).

In 2002, over a decade after the offense, a court-ordered forensic psychiatric evaluation was conducted, formally commissioned by the court during the patient’s prolonged hospitalization in OPG. The report described a chronic psychotic condition marked by affective flattening, rigid thought content, and persecutory ideation. Although some improvement in interpersonal engagement was noted between the two consecutive interviews, the evaluator emphasized the patient’s profound emotional detachment when discussing the homicide. Dangerousness was judged to be attenuated, but its remission was considered conditional on stable psychiatric supervision and continued support from the legal guardian.

Although not formally commissioned by the court, a forensic psychiatric evaluation submitted by the defense in 2010 described G.D.M. as affected by chronic paranoid schizophrenia in clinical remission. The report noted improved affective responsiveness, partial insight into illness, and stable adherence to pharmacological treatment. The expert emphasized the absence of active delusional ideation during the assessment and considered the risk of relapse low, provided that adequate supervision was maintained.

In light of this opinion and the subsequent clinical documentation, the custodial security measure was formally revoked. G.D.M. was authorized to reside with his brother, who had relocated to a different region. This relocation entailed a change in jurisdiction from the original Department of Mental Health to a new service, under which G.D.M. continued to receive outpatient supervision and engage in community-based rehabilitative activities, including regular participation in a psychiatric day center.

In the following years, the patient was gradually transitioned to supervised community placement under a residential rehabilitation program. This trajectory culminated in a fourth forensic psychiatric evaluation in 2014, formally ordered by the court to assess clinical progress and reevaluate the persistence of social dangerousness. The report confirmed a diagnosis of chronic paranoid schizophrenia in stable remission, with partial insight, adequate adherence to treatment, and consistent support from family and services. The forensic expert considered the risk of relapse minimal under maintained supervision and recommended continuation of community-based placement. On this basis, the court formally declared the cessation of social dangerousness, maintaining the subject in a non-custodial but monitored setting. Importantly, these judicial decisions preceded the national closure of OPGs in 2015, so that G.D.M. had already been placed in community care years before that reform.

In 2022, after watching a television documentary on cloning, G.D.M. resumed contact with the victim’s family. He sent unsolicited letters and online messages to the victim’s sister, describing his renewed plans to clone and ‘reunite with’ S.S.

Despite receiving no reply, he persistently attempted further contact through social media platforms (see Supplementary Figure S2).

His conduct prompted new legal action for stalking. A fifth forensic psychiatric evaluation was ordered by the court in 2024, following the resumption of stalking behavior, and was carried out by an appointed expert. He was diagnosed with chronic delusional disorder, with persistent bizarre delusional ideation and sustained lack of insight. Despite over thirty years of clinical, pharmacological, and social interventions, his psychopathology remained largely unchanged. Pharmacological adherence had been consistent, but therapeutic response was poor, suggesting a refractory course. While overt behavioral control had improved, his dangerousness was considered attenuated but still present. He was therefore transferred to a protected residential facility under continued psychiatric care (Table 1).

## 3. Discussion

The clinical and forensic trajectory of G.D.M. highlights longstanding and unresolved challenges within the Italian forensic psychiatric system, especially in the management of chronic, forensic treatment-resistant psychosis. Italy’s model—grounded in the Basaglia Law (Law 180/1978) and the closure of forensic psychiatric hospitals—is internationally recognized for its emphasis on dignity, recovery, and community care [3,5,8,17].

However, it presumes a degree of clinical malleability that does not apply to all psychiatric profiles.

G.D.M.’s persistent delusional ideation, poor insight, and functional impairment underscore the limitations of recovery-based paradigms in cases of enduring psychopathology [18].

His trajectory exemplifies the structural fragility of a system that lacks stratified security levels and long-stay units.

In this regard, a more appropriate long-term solution for a patient with such a chronic, treatment-resistant profile would have been placement in a high-security therapeutic unit specifically designed for long-stay care. Facilities of this kind, available in several European jurisdictions (e.g., the Netherlands), might have offered a more proportionate balance between patient rights, therapeutic opportunities, and sustained risk management, potentially reducing the likelihood of relapse. Unlike other European jurisdictions employing graduated security infrastructures [13,14], Italy relies almost exclusively on REMS facilities, characterized by capped bed availability and limited suitability for individuals with high-risk or non-rehabilitative profiles [3,6].

The case is also notable for its remarkably long interoffense interval—over thirty years—during which G.D.M. was exposed to a wide spectrum of forensic and general psychiatric interventions: hospitalization, supervised release, community-based rehabilitation, and outpatient monitoring. These varied in terms of clinical setting, level of restriction, and continuity of care, reflecting the systemic complexity in managing chronic forensic patients [16]. Yet, the persistent nature of his delusions, especially the unchanged narrative surrounding the victim and cloning, suggests profound treatment resistance. Treatment-resistant psychosis is associated with elevated rates of recidivism and extended stays in secure settings, particularly in the presence of comorbidities such as substance use disorders [19,20].

Although non-pharmacological interventions, notably Cognitive Behavioral Therapy, have shown promise in general offender populations, their effectiveness in forensic psychiatric settings is attenuated by severe psychopathology, institutionalization effects, and unmet criminogenic needs [21,22].

In G.D.M.’s case, the transition from homicide to stalking behavior may suggest a degree of risk mitigation over time. However, the persistence of a fixed delusional system, with unchanged content across decades, indicates a continuing vulnerability to recidivism and a form of enduring psychiatric-based social dangerousness.

His psychopathological structure remained anchored to a delusional nucleus that proved refractory to decades of pharmacological and psychosocial interventions. This partial attenuation of risk—without its elimination—underscores the need for sustained individualized care strategies that address complex criminogenic and psychopathological factors simultaneously.

Several systemic challenges further complicate the management of such cases. These include the absence of validated outcome indicators for forensic psychiatric facilities, the well-documented association between psychopathy severity and recidivism risk [16,23], and the limited efficacy of community-based alternatives such as Community Treatment Orders in patients with poor insight and persistent psychopathology [24].

G.D.M.’s long-term trajectory offers key insights into longitudinal risk management. Periods of close supervision and treatment continuity were associated with stability and behavioral control. Although no direct causal inference can be drawn, episodes of care fragmentation—linked to geographic dislocation, reduced clinical oversight, and the discontinuation of custodial measures—may have contributed to increased long-term vulnerability, with the potential for relapse or recurrence of risk behaviors even after years of apparent stability.

This case also raises critical questions about the ability of DSM lacking adequate and specific training to coordinate effectively the care of forensic patients within a model primarily designed for non-offending individuals. Despite sustained multimodal interventions, the patient did not achieve meaningful insight or reduction in delusional content. His profile remained that of a high-complexity individual, not only due to the chronic nature of his illness but also considering his status as a person who committed a severe, violent crime.

Although the practice historically known as ‘*ergastolo bianco*’—the indefinite detention of mentally ill offenders beyond sentence expiration—has been formally abolished, this case illustrates how the absence of suitable long-term solutions can still result in prolonged containment without true rehabilitative progression.

In fact, while indefinite detention is no longer legally permitted, patients with persistent treatment resistance and enduring risk profiles may remain in the forensic system for many years, as assessments of social dangerousness continue to justify long-term containment.

This is not a repetition of that former legal distortion, but rather a consequence of current systemic gaps, where individuals who committed crimes and do not recover remain suspended in structurally inadequate settings.

As Mandarelli and colleagues (2019) have pointed out, the limitations of a recovery-based model become particularly evident when confronted with therapeutic exhaustion and chronicity [15].

In this regard, the present reconstruction—based on clinical and forensic records spanning over three decades, produced in different contexts and with variable diagnostic approaches and data completeness—also presents inherent limitations.

A further limitation of the present reconstruction is the lack of standardized psychometric data over the thirty-year trajectory. Apart from the Rorschach test administered in the earliest forensic evaluation, no systematic assessments of IQ, substance use, cognitive decline, or structured risk instruments (e.g., PCL-R) were available. This highlights the importance of consistently implementing standardized tools in forensic psychiatric practice.

While the persistence of psychopathology over time strongly suggests treatment resistance, incomplete documentation of the therapeutic pathways, especially during earlier hospitalizations, means this can only be inferred rather than conclusively demonstrated.

Structured risk assessment protocols and long-term management strategies, particularly for patients with a history of violent acts, remain essential tools [3,25,26]

Rather than representing an anomaly, G.D.M.’s case reflects a broader structural blind spot in Italian forensic psychiatry. Without the development of secure long-stay facilities, high-security therapeutic units, and integrated post-REMS pathways within DSM networks, the system will continue to fail those who fall outside its rehabilitative logic.

Addressing these gaps requires political and institutional commitment to develop high-security therapeutic units, implement routine outcome monitoring, health professionals’ adequate and specific training and ensure robust legal safeguards, thereby reconciling patient rights with public safety.

His trajectory calls for renewed institutional and political commitment to secure, ethically sound, and clinically appropriate alternatives for patients who, although stabilized in part, remain non-rehabilitated and at ongoing forensic risk.

## 4. Conclusions

This case highlights enduring tensions within Italy’s right-based forensic psychiatric system, particularly in the management of individuals with treatment-resistant disorders and a history of serious violence.

In the absence of stratified levels of therapeutic security, structured post-REMS solutions, and a clear redefinition of the role of Mental Health Departments in the management of forensic patients, these individuals risk becoming trapped in prolonged supervisory arrangements that are neither legally clear nor clinically effective.

G.D.M.’s trajectory highlights the need for proportionate, individualized responses to cases that deviate from standard rehabilitative pathways. Psychiatric patients who have committed serious offenses often present persistent clinical and forensic complexity, requiring long-term strategies that go beyond community-based models alone.

Policymakers should prioritize the development of secure long-stay therapeutic facilities, the implementation of routine outcome monitoring, health professionals’ adequate and specific training and the strengthening of legal safeguards to ensure that the system can balance individual rights with public protection, ethically, clinically, and institutionally.

## Figures and Tables

**Table 1:** Chronological timeline of key clinical and forensic events in the case of G.D.M. (1988–2024).

Year	Age	Key Event	Clinical/Forensic Outcome
1988	30	Initial police report following persistent stalking behavior	Outpatient psychiatric assessment; provisional diagnosis of schizophrenia; antipsychotic treatment proposed but refused
1989	31	Physical assault on the victim	Second complaint filed; haloperidol decanoate initiated but discontinued due to extrapyramidal side effects
1991	33	Homicide of the victim and subsequent forensic evaluation	Diagnosis of chronic schizophrenia; total criminal irresponsibility; high social dangerousness; admission to OPG
2001	43	Decennial forensic review	Transfer to a supervised residential rehabilitation facility (medium security level)
2002	44	Court-ordered forensic reassessment during OPG placement	Persistence of chronic psychosis with flattened affect and poor insight; dangerousness considered absent under supervision
2010	52	Judicial forensic re-evaluation	Paranoid schizophrenia with partial insight; dangerousness considered attenuated; cohabitation with brother authorized
2014	54	New court-ordered forensic evaluation to reassess dangerousness	Stable remission under continued therapy; partial awareness of illness; dangerousness declared ceased by judicial authority
2022	64	Resumption of delusional contact with the victim’s sister	New stalking charges filed; documentation of partial antipsychotic non-adherence
2024	66	Forensic psychiatric evaluation following recurrence of threatening behavior	Chronic delusional disorder; persistent lack of insight; dangerousness considered attenuated but not absent; admission to protected facility

Note. OPG: forensic psychiatric hospitals (Ospedali Psichiatrici Giudiziari), former custodial institutions for mentally ill offenders in Italy. OPGs were formally abolished in 2015 following national psychiatric reform and replaced by REMS (Residences for the Execution of Security Measures).

## Data Availability

No additional data are available due to the highly sensitive and confidential nature of the clinical and forensic information contained in this case report.

## Supplementary Materials

The following supporting information can be downloaded at: https://www.mdpi.com/article/10.3390/healthcare13182302/s1, Figure S1: Letter sent by G.D.M. via post in 1996 to the victim’s mother of S.S.; Figure S2: Letter sent by G.D.M. via post in the summer of 2022 to the victim’s namesake cousin (S.S.), subsequently forwarded by email to the sister of S.S.
