# Supplementary material for: A Thirty-Year Forensic Case Report of Chronic Psychosis: Systemic Implications for Italian Forensic Psychiatry
Source: Healthcare (Basel). 2025 Sep 15;13(18):2302. doi: 10.3390/healthcare13182302 (PMC12469453; doi:10.3390/healthcare13182302)
Supplement: Supplementary file 1 [file healthcare-13-02302-s001.zip › healthcare-3831805-supplementary.pdf]

**Figure S1.** Letter sent by G.D.M. via post in 1996 to the victim's mother of S.S.

IO, [redacted], DICHIARO QUANTO SEGUE: 3.

ESISTE UNA REALE POSSIBILITÀ DI FAR RINASCERE
LA STESSA PERSONA DECEDUTA, CON UNI PROCEDIMENTO
DI INGEGNERIA GENETICA. QUESTO PROCEDIMENTO È
SEGRETO, NESSUNO LO CONOSCE, CONSISTE IN OPERAZIONI
BEN PRECISE. IL COSTO È NELL'ORDINE DEI 50 MILIONI
DI LIRE CIRCA. IL MIO AMORE PER [redacted] LO HA
REALIZZATO.

SI ESTRAE DAL CORPO DELLA MADRE DEL DEFUNTO UN
NUMERO ELEVATO DI CELLULE UOVO (SONO QUELLE CHE
HANNO 23 CROMOSOMI) ADATTE ALLA FECONDAZIONE
IN VITRO, SE DALLA MADRE NON È POSSIBILE PERCHÉ
DECEDUTA O LE CELLULE UOVO NON SONO UTILIZZABILI
PER LA FECONDAZIONE, QUESTE CELLULE SI PRENDONO
DA UNA SORELLA DEL DEFUNTO. È NECESSARIO CHE SI
RICREI L'AMBIENTE ORIGINARIO, QUINDI I RIBOSOMI
MATERNI DEVONO ESSERE TRAPIANTATI NEL CITOPLASMA
DELLA CELLULA UOVO DELLA SORELLA DEL DEFUNTO AL
POSTO DEI RIBOSOMI NATURALMENTE PRESENTI,
QUESTO NELL'IPOTESI CHE NON SI POSSONO UTILIZZARE
LE CELLULE UOVO MATERNE.
IL DNA PRESO DAI RESTI DELLE OSSA DEL DEFUNTO,
DEVE ESSERE PERFETTO, CIOÈ NON VI DEVE ESSERE
FRAMMENTAZIONE DELLA DOPPIA ELICA DEL DNA, QUINDI
BISOGNA ANALIZZARLO, PURIFICARLO, PREPARARLO.
POICHÉ BISOGNA RICREARE L'AMBIENTE ORIGINARIO,
BISOGNA DIVIDERE I CROMOSOMI DI ORIGINE PATERNA
DA QUELLI DI ORIGINE MATERNA PER RICONGIUNGERLI
E ATTIVARE LA MITOSI. PER QUESTO BISOGNA PRELEVARE
DNA DAL PADRE E DALLA MADRE E CONFRONTARLO
CON QUELLO DEL DEFUNTO. UNA VOLTA SAPUTO QUAL'È,

SI PROCEDE ALL'OPERAZIONE DI ESTRAZIONE
DEL DNA DELLA CELLULA UOVO SELEZIONATA
IN MODO CHE AL SUO POSTO SIA INTRODOTTO
IL DNA DEL DECEDUTO DI ORIGINE MATERNA
(23 CROMOSOMI). QUESTO PERCHÉ IL DNA
DEL FIGLIO PUR VENENDO DAL DNA MATERNO
NON È PIÙ LO STESSO, IN QUANTO HA SUBITO DELLE
MODIFICAZIONI NELLO SCAMBIO GENICO CON IL
DNA PATERNO. DAL PADRE SI PRELEVANO
CELLULE GERMINALI (SPERMATOZOI) E SI SOSTITUISCE
IL DNA DI UNO SPERMATOZOO CON IL DNA DEL
FIGLIO DI ORIGINE PATERNA CIOÈ GLI ALTRI 23
CROMOSOMI. QUINDI, IN VITRO, SI FECONDA
LA CELLULA UOVO INTRODUCENDO LO SPERMATOZOO
E CIÒ CHE CI ASPETTIAMO È CHE INIZIA
LA MITOSI CHE PORTERÀ ALLA NASCITA DEL
DECEDUTO. BISOGNA CORREGGERE GLI ERRORI
SEMPRE IN VISTA DELLA MITOSI.
QUESTO NELLE LINEE GENERALI È IL PROCEDIMENTO.
LE DIFFICOLTÀ POSSONO ESSERE SUPERATE CON
L'INTERVENTO DI SPECIALISTI IN INGEGNERIA GENETICA.
IL PARTO DEVE FARLO LA SORELLA DEL DEFUNTO,
IN OGNI CASO CONTROLLARE CHE IL DNA DELL'EMBRIONE
SIA QUELLO DEL DECEDUTO, VICEVERSA ABORTIRE
GIÀ IN VITRO, CIOÈ PRIMA DELL'IMPIANTO IN
UTERO, E RIPETERE LE OPERAZIONI CORREGGENDO
GLI ERRORI.
CON QUALSIASI ESPERTO PARLERETE,
VI DIRÀ CHE NON È POSSIBILE FARLO,
DOVETE RISCHIARE, È UNA NOVITÀ ASSOLUTA.

SE IL DNA NON ATTIVA LA
MITOSI E IL PROBLEMA DIPENDE
DAI GENI CHE SONO SPENTI,
IL PROCEDIMENTO DIVENTA
LUNGO, DIFFICILE E COSTOSO,
INFATTI SU OGNI CROMOSOMA
POSSONO ESSERCI CIRCA 2500 GENI
E QUINDI È UMANAMENTE
IMPROPONIBILE UN LAVORO DI
COPIATURA DI 100'000 GENI
A MENO CHE QUESTO LAVORO
SIA FATTO DA MACCHINE ELETTR
CHIMICHE APPOSITAMENTE
COSTRUITE PER QUESTI LAVORI:
LE NANOMACCHINE STM.

LA MIA MISSIONE TERMINA QUI,
NON HO PIÙ NULLA DA DIRVI.

FIRMA

11-5-1996 [redacted]

**Note.** Literal translation: '*There is a real possibility to bring back to life the same deceased person, with a genetic engineering procedure. This procedure is secret, no one knows it, it consists of very precise operations. My love for Santa has realized it. A large number of egg cells (those that have 23 chromosomes) suitable for in vitro fertilization are extracted from the body of the deceased's mother; if it is not possible from the mother because she is deceased or the egg cells are not usable for fertilization, these cells are taken from a sister of the deceased. It is necessary to recreate the original environment, so the maternal ribosomes must be transplanted into the cytoplasm of the egg cell of the deceased's sister in place of the naturally present ribosomes; this in the hypothesis that maternal egg cells cannot be used. The DNA taken from the remains of the deceased's bones must be perfect, that is, there must be no fragmentation of the DNA double helix, so it must be analyzed, purified, prepared. Since the original environment must be recreated, the chromosomes of paternal origin must be separated from those of maternal origin to rejoin them and activate mitosis. For this, DNA must be taken from the father and mother and compared with that of the deceased, once it is known which it is. The operation of extracting the DNA of the selected egg cell is carried out so that the DNA of maternal origin of the deceased (23 chromosomes) is introduced in its place. This is because the child's DNA coming from the maternal DNA is no longer the same, as it has undergone modifications in the gene exchange with the paternal DNA. Germ cells (sperm) are taken from the father and the DNA of one sperm is replaced with the DNA of the child of paternal origin, that is, the other 23 chromosomes. Then, in vitro, the egg cell is fertilized by introducing the sperm and what we expect is that mitosis begins which will lead to the birth of the deceased. Errors must always be corrected with mitosis in mind. This is the general procedure. Difficulties can be overcome with the intervention of specialists in genetic engineering. The birth must be done by the sister of the deceased; in any case, check that the DNA of the embryo is that of the deceased, otherwise abort already in vitro, that is, before implantation in the uterus, and repeat the operations correcting the errors. Any expert you talk to will tell you that it is not possible*

*to do it, you must take the risk, it is an absolute novelty. If the DNA does not activate mitosis and the problem depends on genes that are turned off, the procedure becomes long, difficult and expensive; in fact, on each chromosome there can be about 2500 genes and therefore it is humanly unfeasible to copy 100,000 genes unless this work is done by electric chemical machines specially built for these tasks: the STM nanomachines. My mission ends here, I have nothing more to tell you.'*

**Figure S2.** Letter sent by G.D.M. via post in the summer of 2022 to the victim's namesake cousin (S.S.), subsequently forwarded by email to the sister of S.S.

united states
synthetic biology laboratories
dott george church

vi prego di dare questa lettera a [redacted]
la sorella di [redacted]: [redacted]

[redacted] questo professore hà la capacità di
far rinascere qualsiasi essere
partendo dal DNA delle cellule delle ossa.
il parto può avvenire in una nazione dove è ammesa la maternità surrogata.

carissima sorella di [redacted]
sento il bisogno di chiarire quanto segue:

[redacted] ha ricevuto solo 4 coltellate e non 14,
le prime due davanti e le altre due alla schiena quando
ha tentato di fuggire,a quel punto la lama del coltello
si è piegata a fisarmonica avento urtato un osso
rendendomi impossibile continuare a colpirla.

Credete pure che [redacted] sia stata uccisa da un pazzo
schizofrenico indemoniato,
ma la realtà potrebbe essere diversa.
Se [redacted] mi avesse pregato di non ucciderla,quella sera,
non l'avrei uccisa.
La preghiera fa miracoli.

Non ti dico i guai che ho passato e stò passando
con questi cretini di psichiatri che impongono
sostanze che chiamano farmaci ma sono di una
tossicità estrema e che fanno stare male e non bene.

se decidi di contattare il proff church per far
rinascere [redacted] sappi che troverai grossi problemi
nella legislazione americana che proibisce la
clonazione e sicuramente il proff non vorrà
andare in galera per aver clonato [redacted]
quindi aspettati un no da parte sua.dovrai pregarlo.
l'unica strada è la segretezza dell'operazione.
Si potrebbe spostare la macchina clonatrice in
una nazione permissiva e così risolvere il problema.

Ma prima di tutto chiediti : vale la pena far
rinascere [redacted] in questo mondo di stronzi ?

George M. Church

Harvard Medical School
Department of Genetics
Nee Research Building, Room 238
77 Avenue Louis Pasteur
Boston, MA 02115
gchurch@genetics.med.harvard.edu
p: 617 432-1278
https://arep.med.harvard.edu/visit.html

church_lab_admin@hms.harvard.edu

**Note.** Literal translation: *'United States. Synthetic biology laboratories. dott. george church. Please give this letter to *** sister of S.S,: this professor has the ability to bring any being back to life starting from the DNA of bone cells. The birth can take place in a country where surrogate motherhood is allowed. Dearest sister of S.S. I feel the need to clarify the following: S.S. received only 4 stab wounds and not 14, the first two in the front and the other two in the back when she tried to escape, at that point the knife blade folded like an accordion after hitting a bone making it impossible for me to continue stabbing her. You may believe that S.S. was killed by a crazy schizophrenic, demon-possessed madman, but the reality could be different. if S.S. had begged me not to kill her, that night, I would not have killed her. Prayer works miracles. I won't tell you the troubles I have gone through and am still going through with these idiot psychiatrists who impose substances they call medicines but are extremely toxic and make people feel worse, not better. If*

*you decide to contact Prof. Church to bring S.S. back to life, know that you will encounter big problems with American legislation, which prohibits cloning and surely the Prof. will not want to go to jail for having cloned S.S. so expect a 'no' from him. You will have to pray to him. The only way is the secrecy of the operation. One could move the cloning machine to a permissive country and thus solve the problem. But first of all ask yourself: is it worth bringing S.S. back to life in this world of assholes? George M. Church. Harvard Medical School. Department of Genetics. Nee Research Building Room 238. 77 Avenue Louis Pasteur. Boston, MA 02115. gchurch@genetics.med.harvard.edu. P: 617-432-1278. https:// arep.med.harvard.edu/visit.html. churh_lab_admin@hms.harvard.edu'*
